# Predicting severe renal dysfunction in alcohol-associated cirrhosis: Comparative performance of liver function scores and machine learning models

**DOI:** 10.1371/journal.pone.0332840

**Published:** 2025-09-17

**Authors:** Julian Müller-Kühnle, Moritz Schanz, Severin Schricker, Christian Benignus, Julia Todoroff, Jörg Latus, Wolfram Zoller, Dominik Marschner

**Affiliations:** 1 Department of General Internal Medicine and Nephrology, Robert Bosch Hospital, Stuttgart, Germany; 2 Robert Bosch Society for Medical Research, Stuttgart, Germany; 3 Paracelsus Medical University, Salzburg, Austria; 4 Department of General, Visceral, Thoracic, and Pediatric Surgery, Ludwigsburg Hospital, Ludwigsburg, Germany; 5 Department of Hematology, Oncology, Stem Cell Transplantation, and Palliative Medicine, Klinikum Stuttgart, Stuttgart, Germany; 6 Department of General Internal Medicine, Gastroenterology, Gastrointestinal Oncology, Hepatology, Infectious Diseases, and Pulmonology, Klinikum Stuttgart, Stuttgart, Germany; Shaanxi Provincial People's Hospital, CHINA

## Abstract

**Background:**

Renal dysfunction is a frequent and clinically relevant complication of cirrhosis, yet chronic kidney disease (CKD) often remains underrecognized, particularly in non-acute settings. Early identification of at-risk patients is essential to guide timely interventions. Although MELD, Child-Pugh Score (CPS), APRI, and FIB-4 are widely used to assess hepatic disease severity, their predictive value for advanced renal dysfunction is uncertain.

**Methods:**

In this retrospective cohort study (2014–2021, Klinikum Stuttgart), we evaluated the ability of MELD, CPS, APRI, and FIB-4 to predict severe renal dysfunction (chronic kidney disease [CKD] stage ≥ 3, according to Kidney Disease: Improving Global Outcomes [KDIGO] classification) in patients with alcoholic cirrhosis. Logistic and linear regression analyses were performed. In addition, machine learning (ML) models were trained to identify non-renal predictors of CKD stage ≥ 3.

**Results:**

Among 131 patients (mean age 62.8 ± 11.3 years; 71% male), 33% met criteria for KDIGO stage ≥ 3. MELD was significantly associated with advanced CKD (OR = 1.379, p < 0.001), with prevalence increasing from 17% (MELD ≤ 9) to 80% (MELD ≥ 20). CPS showed an inverse association (p = 0.002), while APRI and FIB-4 were not predictive. The optimized Random Forest model, refined through ROSE oversampling and feature selection, achieved an AUC of 0.757, with 76% accuracy, 82% sensitivity (KDIGO < 3), and 63% specificity (KDIGO ≥ 3).

**Conclusion:**

MELD was the most reliable conventional score for identifying advanced renal dysfunction in alcoholic cirrhosis. ML-based models incorporating routinely available clinical parameters further improved predictive performance and may support risk stratification in this high-risk population.

## Introduction

Chronic kidney disease (CKD) is a frequent and underrecognized complication of liver cirrhosis, contributing to increased morbidity, hospitalizations, and mortality [1–4]. While renal dysfunction in acute settings – such as hepatorenal syndrome (HRS) – has been extensively studied, early detection of chronic impairment remains challenging, particularly in compensated or non-acute disease stages [5–7]. Alcohol-associated cirrhosis, which continues to represent a major global burden, is especially prone to renal complications owing to its frequent coexistence with malnutrition, systemic inflammation, and circulatory dysfunction [8–11].

Given the prognostic relevance of renal impairment, reliable risk stratification tools are needed. Widely used hepatic scores, such as the Model for End-Stage Liver Disease (MELD) and Child-Pugh Score (CPS), are applied to assess liver disease severity and prioritize transplant allocation [12–15]. MELD incorporates serum creatinine and has been linked to renal dysfunction and mortality [16,17]. In contrast, CPS and fibrosis-based indices such as the AST-to-Platelet Ratio Index (APRI) and Fibrosis-4 Score (FIB-4) – developed for staging fibrosis or predicting survival – do not include renal parameters and may be less suitable for identifying patients at risk of CKD [18–21].

Moreover, serum creatinine is an imperfect marker in cirrhosis due to confounding from muscle wasting and altered hepatic metabolism, particularly in sarcopenic or malnourished individuals [22–24]. These limitations underline the need for novel, data-driven approaches that incorporate broader clinical and biochemical features.

In this study, we systematically evaluated the performance of four conventional liver scores – MELD, CPS, APRI, and FIB-4 – for predicting advanced chronic kidney disease (CKD; stage ≥ 3 according to Kidney Disease: Improving Global Outcomes [KDIGO] classification) in patients with alcoholic cirrhosis. We also applied supervised machine learning (ML) methods to identify supplementary, non-renal predictors of renal dysfunction. While the focus is on alcohol-associated disease, the modeling approach may be transferable to other etiologies with appropriate recalibration. The overarching goal was to improve renal risk stratification using both established clinical tools and modern data analytics. By directly addressing the limited representation of renal dysfunction in widely used hepatic scores, this study aims to close an important gap in current risk stratification approaches. Specifically, we not only compare conventional scores with CKD outcomes but also evaluate novel, non-renal predictors through ML to provide a broader framework for early risk assessment.

## Methods

### Study design and population

This retrospective cohort study was conducted at Klinikum Stuttgart, a tertiary care center in Germany, between 2014 and 2021. The primary objective was to assess the predictive performance of four commonly used liver scores – MELD, CPS, APRI, and FIB-4 – identifying advanced renal dysfunction, defined as chronic kidney disease (CKD) stage ≥ 3 according to KDIGO guidelines. In addition, supervised ML models were applied to identify non-traditional predictors beyond hepatic function.

Eligible participants were adults (≥18 years) with clinically and radiologically confirmed alcoholic cirrhosis and a documented history of chronic alcohol use. Endoscopic and ultrasonographic examinations were performed within one week before or after hospital admission to ensure consistent staging. Laboratory values, including serum creatinine and estimated glomerular filtration rate (eGFR), were obtained at admission.

Exclusion criteria were acute kidney injury (AKI) according to KDIGO guidelines, unstable renal function prior to baseline, and cirrhosis of non-alcoholic etiology (e.g., viral hepatitis, autoimmune, or cholestatic liver disease). Common comorbidities such as diabetes mellitus and arterial hypertension were not excluded to preserve external validity. Patients with substantial missing data were excluded unless the extent of missingness was low and could be addressed by imputation (see below).

### Evaluation of renal function and hepatic disease severity

Renal function was assessed using eGFR, calculated with the Chronic Kidney Disease Epidemiology Collaboration (CKD-EPI) formula. CKD stage ≥ 3 was defined as eGFR < 60 mL/min/1.73 m² in accordance with KDIGO staging.

Liver disease severity was evaluated using four established scoring systems: MELD, CPS, APRI, and FIB-4. These scores were selected because they are widely applied in routine clinical practice and represent complementary aspects of hepatic disease severity and fibrosis, while enabling assessment of their potential utility for predicting renal dysfunction.

### Statistical analysis

Patients were stratified into two groups: KDIGO stage < 3 and KDIGO stage ≥ 3. Continuous variables were tested for normality and compared using analysis of variance (ANOVA) or Kruskal–Wallis tests, as appropriate. Categorical variables were analyzed with chi-square or Fisher’s exact tests. Correlations between liver scores and renal markers were assessed using Spearman’s rank correlation.

Binary logistic regression was used to examine associations between liver scores and the likelihood of KDIGO stage ≥ 3. Linear regression models were applied to evaluate the relationship between liver scores and eGFR as a continuous variable. Statistical significance was defined as p ≤ 0.05.

Given the exploratory nature of this study, no adjustment for multiple comparisons was applied. In line with recommendations by Bender and Lange [39] and Streiner [40], p-values are interpreted descriptively and are not used for confirmatory inference.

### Machine learning-based prediction of severe renal dysfunction

To identify non-renal predictors of KDIGO stage ≥ 3, several ML models were developed. Variables directly linked to renal function (e.g., creatinine, urea, eGFR) were excluded to avoid circular reasoning. Candidate features were selected based on clinical relevance, data availability, and prior literature. The final feature set included age, sex, body mass index (BMI), international normalized ratio (INR), alanine aminotransferase (ALT), platelet count, bilirubin, presence and grade of hepatic encephalopathy, ascites volume, and spleen diameter.

An initial Random Forest (RF) model was trained using 1,000 trees. To improve performance, a refined model with 50,000 trees was built, incorporating hyperparameter tuning (mtry = 3), class weighting, and oversampling with the Random OverSampling Examples (ROSE) method [25]. ROSE was chosen over the Synthetic Minority Oversampling Technique (SMOTE) because it natively handles mixed-type data without requiring adaptations such as SMOTE for Nominal and Continuous (SMOTE-NC) variables [26].

Model performance was evaluated using accuracy, sensitivity, specificity, balanced accuracy, area under the receiver operating characteristic curve (AUC), and Cohen’s kappa. Feature importance was quantified using mean decrease in Gini impurity.

Comparative XGBoost models [27] were trained using 500 and 5,000 trees with early stopping. These models, however, exhibited severe overfitting and poor external performance (AUC < 0.5) and were therefore excluded from final evaluation.

### Missing data and imputation

Minor missingness was present for selected variables (e.g., ALT, bilirubin, spleen diameter). For ML models, missing values were imputed within KDIGO strata using the median (continuous variables) or mode (categorical variables). Conventional statistical analyses were performed on complete-case data.

### Ethics statement

This study was conducted in accordance with institutional and data protection regulations. Ethical review was waived by the Ethics Committee of the University of Tübingen (Reference 607/2021BO2) in accordance with § 27 Bundesdatenschutzgesetz (BDSG) and Articles 5, 6, 9, and 89 of the General Data Protection Regulation (GDPR). Informed consent was not required due to the retrospective and anonymized study design. To ensure compliance, data extraction and analysis were performed by different individuals.

### Software and tools

All classical statistical analyses were performed using the Statistical Package for the Social Sciences (SPSS) version 29.0 (IBM Corp., Armonk, NY, USA). ML modeling and receiver operating characteristic (ROC) analysis were conducted in R version 4.4.2 (R Foundation for Statistical Computing, Vienna, Austria).

## Results

### Study population

A total of 131 patients with alcoholic liver cirrhosis were included. The mean age was 62.8 ± 11.3 years, and 71% were male. The average body mass index (BMI) was 25.7 ± 2.6 kg/m². Ascites was present in 71% of patients, and hepatic encephalopathy in 23%. The mean portal vein velocity was 17.7 ± 6.1 cm/s, and the mean spleen diameter was 130.3 ± 25.8 mm.

Renal parameters revealed a mean serum creatinine of 1.2 ± 0.8 mg/dL, an estimated glomerular filtration rate (eGFR) of 78.6 ± 33.7 mL/min, and a mean urea level of 71.6 ± 36.5 mg/dL. According to Kidney Disease: Improving Global Outcomes (KDIGO) criteria, 43 patients (33%) met the definition of CKD stage ≥ 3.

Baseline demographics, laboratory values, and prognostic scores are summarized in Table 1. Detailed endoscopic findings, including variceal grading and intervention requirements, are provided in Supplementary Table S1.

**Table 1:** Clinical, laboratory, and endoscopic characteristics of the study cohort, stratified by KDIGO stage < 3 and ≥ 3.

	Total (n = 131)	KDIGO < 3 (n = 88)	KDIGO ≥ 3 (n = 43)	p	Effect size
**Demographics**					
Age (years)	62.8 ± 11,3	60.5 ± 11.3	**67.6 ± 10.5**	**0.001**	−0.65
BMI (kg/m²)	25.7 ± 2.6	25,9 ± 2.5	25.5 ± 2.9	0.4	0.16
Gender Distribution (M/F; %)	93 M (70.9%), 38F (29.1%)	62M (70.5%), 26F (29.5%)	31 M (72.1%), 12 F (27.9%)	0.85	0.02
**Laboratory parameters**					
Creatinine (mg/dl)	1.2 ± 0.8	0.8 ± 0.2	**2 ± 0.9**	**0.001**	−2.3
eGFR (ml/min/1.73m²)	78.6 ± 33.7	98.4 ± 19.8	**36.6 ± 13.6**	**0.001**	3.4
Urea (mg/dl)	71.6 ± 36.5	46.7 ± 26.4	**98.2 ± 28.2**	**0.002**	−1.25
Hemoglobin (g/dL)	10.4 ± 2.6	10.6 ± 2.6	9.8 ± 2.4	0.09	0.32
AST (U/l)	84.2 ± 79.6	93.9 ± 88.5	62.9 ± 45.6	0.06	0.4
ALT (U/l)	42.8 ± 35.5	44.5 ± 33.6	39 ± 39.3	0.41	0.15
Platelets (10³/µL)	131.9 ± 94.4	129.1 ± 95.4	137.7 ± 93.8	0.63	−0.09
Total bilirubin (md/dl)	3.4 ± 5	3.5 ± 5	3.3 ± 4.8	0.79	0.05
INR	1.5 ± 0.4	1.5 ± 0.4	1.4 ± 0.3	0.47	0.01
**Prognostic Scores**					
CPS	8.9 ± 1.8	9.1 ± 1.9	8.5 ± 1.7	0.09	0.33
MELD	12.1 ± 4.3	11 ± 3.5	**14.5 ± 5**	**0.001**	−0.85
APRI	3.6 ± 7.1	4.2 ± 8.1	2.5 ± 2.3	0.11	0.24
FIB4	11.2 ± 11.8	12.2 ± 12.9	8.8 ± 6.3	0.15	0.24

Continuous variables are presented as mean ± standard deviation; categorical variables as percentages. Effect sizes are reported as standardized mean differences (φ). Statistically significant results (p ≤ 0.05) are shown in bold. Detailed endoscopic findings are provided in Supplementary Table S1.

Abbreviations: KDIGO, Kidney Disease: Improving Global Outcomes; BMI, body mass index; AST, aspartate aminotransferase; ALT, alanine aminotransferase; INR, international normalized ratio; CPS, Child-Pugh score; MELD, Model for End-stage Liver Disease; APRI, aspartate aminotransferase-to-platelet ratio index; FIB-4, fibrosis-4 index.

### Comparison of KDIGO < 3 and KDIGO ≥ 3 Groups

Patients with KDIGO stage ≥ 3 were significantly older than those with KDIGO < 3 (67.6 ± 10.5 vs. 60.5 ± 11.3 years; p = 0.001, φ = −0.65). They also showed higher serum creatinine (2.0 ± 0.9 vs. 0.8 ± 0.2 mg/dL; p = 0.001, φ = −2.3), lower eGFR (36.6 ± 13.6 vs. 98.4 ± 19.8 mL/min; p = 0.001, φ = 3.4), and elevated urea levels (98.2 ± 28.2 vs. 46.7 ± 26.4 mg/dL; p = 0.002, φ = −1.25).

There was a trend toward lower hemoglobin (9.8 ± 2.4 vs. 10.6 ± 2.6 g/dL; p = 0.09) and lower AST (62.9 ± 45.6 vs. 93.9 ± 88.5 U/L; p = 0.06) in the KDIGO ≥ 3 group. No significant differences were observed for platelet count, INR, ALT, or total bilirubin.

While the overall prevalence of varices did not differ significantly, Paquet grade 3 varices were exclusively observed in the KDIGO ≥ 3 group (5%; p = 0.04, φ = 0.18; Supplementary S1 Table). Endoscopic interventions were also more common in this group (44% vs. 27%; p = 0.05, φ = 0.17).

MELD scores were significantly higher in patients with KDIGO ≥ 3 (14.5 ± 5.0 vs. 11.0 ± 3.5; p = 0.001, φ = −0.85), whereas CPS, APRI, and FIB-4 did not differ significantly between groups (Table 1).

### Associations between liver function scores and renal dysfunction

A stepwise increase in the prevalence of KDIGO stage ≥ 3 was observed with higher MELD strata: 17% (MELD ≤ 9), 36% (MELD 10–19), and 80% (MELD ≥ 20) (p = 0.002; Table 2). Corresponding changes in renal parameters included increasing serum creatinine (0.9 ± 0.5 to 2.7 ± 1.4 mg/dL) and decreasing eGFR (88.3 ± 26.2 to 35.5 ± 35.3 mL/min) (both p < 0.001). Urea levels followed a similar pattern (p < 0.001).

**Table 2:** Renal function parameters across MELD and FIB-4 strata.

	MELD ≤ 9 (n = 24)	MELD 10–19 (n = 73)	MELD ≥ 20 (n = 10)	p	FIB-4 < 3.25 (n = 15)	FIB-4 ≥ 3.25 (n = 81)	p
**KDIGO STAGE**							
KDIGO < 3	20 (83.3%)	47 (64.4%)	2 (20%)	**0.002**	11 (73.3%)	55 (67.9%)	0.68
KDIGO ≥ 3	4 (16.7%)	26 (35.6%)	8 (80%)		4 (26.7%)	26 (32.1%)	
**Laboratory parameters**							
Creatinine (mg/dL)	0.9 ± 0.5 [0.76-1.04]	1.1 ± 0.5 [0.96-1.24]	**2.7 ± 1.4 [1.69-3.74]**	**<0.001**	1 ± 0.4 [0.74-1.26]	1.1 ± 0.7 [0.94-1.26]	0.46
eGFR (mL/min/1.73 m²)	88.3 ± 26.2 [79.7-96.9]	78.2 ± 34.4 [71.1-85.2]	**35.5 ± 35.3 [9.2-61.8]**	**<0.001**	84.8 ± 31.6 [71.0-98.6]	78.7 ± 33.1 [72.0-85.5]	0.52
Urea (mg/dL)	61.4 ± 40.6 [35.6-87.2]	60.3 ± 39.9 [43.0-77.5]	**146.8 ± 63.2 [68.3-225.3]**	**<0.001**	32.8 ± 26.8 [12.1-53.6]	63.8 ± 38 [52.0-75.6]	0.13

Continuous variables are presented as mean ± standard deviation with 95% confidence intervals in brackets. Categorical variables are shown as counts and percentages. Statistically significant differences (p ≤ 0.05) are shown in bold.

Abbreviations: MELD, Model for End-stage Liver Disease; FIB-4, Fibrosis-4 index; KDIGO, Kidney Disease: Improving Global Outcomes; eGFR, estimated glomerular filtration rate.

No significant differences in renal markers were observed across CPS, APRI, or FIB-4 strata (all p > 0.05), although a trend toward lower eGFR with higher CPS values was noted (p = 0.05; Table 3).

**Table 3:** Renal function markers and KDIGO stage distribution across Child-Pugh score (CPS) and APRI strata.

	CPS A (n = 26)	CPS B (n = 67)	CPS C (n = 38)	p	APRI < 2 (n = 49)	APRI ≥ 2 (n = 49)	p
**KDIGO Stage**							
KDIGO < 3	17 (65.4%)	41 (61.2%)	30 (78.9%)	0.17	31 (63.3%)	36 (73.5%)	0.28
KDIGO ≥ 3	9 (34.6%)	26 (38.8%)	8 (21.1%)		18 (36.7%)	13 (26.5%)	
**Laboratory parameters**							
Creatinine (mg/dL)	1 ± 0.5 [0.76-1.26]	1.2 ± 0.9 [0.96-1.44]	1.1 ± 0.7 [0.86-1.34]	0.33	1.1 ± 0.6 [0.95-1.25]	1.1 ± 0.7 [0.94-1.26]	0.76
eGFR (mL/min/1.73 m²)	84.9 ± 34.3 [71.0-98.7]	70.7 ± 32.7 [62.7-78.6]	85.9 ± 34.9 [74.4-97.4]	0.05	76.3 ± 32.2 [66.2-86.3]	82.4 ± 33.1 [72.9-91.9]	0.37
Urea (mg/dL)	39.8 ± 22.4 [12.0-67.6]	80 ± 59.1 [56.0-104.4]	63.1 ± 30.7 [42.5-83.7]	0.23	60.4 ± 39.3 [42.0-78.8]	59.4 ± 37.1 [36.9-81.8]	0.94

Continuous variables are presented as mean ± standard deviation with 95% confidence intervals in brackets. Categorical variables are shown as counts and percentages. Statistically significant differences (p ≤ 0.05) are shown in bold. A borderline trend in estimated glomerular filtration rate (eGFR) across CPS categories was observed (p = 0.05).

Abbreviations: KDIGO, Kidney Disease: Improving Global Outcomes; CPS, Child-Pugh score; APRI, aspartate aminotransferase-to-platelet ratio index; eGFR, estimated glomerular filtration rate.

### Correlation analyses

Among all liver scores, MELD showed the strongest associations with renal parameters: a positive correlation with serum creatinine (r = 0.333; p < 0.001) and a negative correlation with eGFR (r = −0.294; p = 0.001). The correlation between MELD and urea did not reach statistical significance (r = 0.282; p = 0.078). No significant correlations were found for APRI, CPS, or FIB-4 with any renal marker (Table 4).

**Table 4:** Correlation between liver function scores and renal function parameters (Spearman’s rank correlation).

Variable 1	Variable 2	r	p
**MELD**	Creatinine	0.333	**< 0.001**
**MELD**	GFR	**−0.294**	**0.001**
**MELD**	Urea	0.282	0.078
**APRI**	Creatinine	−0.083	0.416
**APRI**	GFR	0.081	0.436
**APRI**	Urea	0.037	0.838
**CPS**	Creatinine	−0.029	0.740
**CPS**	GFR	0.045	0.612
**CPS**	Urea	0.092	0.568
**FIB-4**	Creatinine	−0.044	0.668
**FIB-4**	GFR	0.026	0.804
**FIB-4**	Urea	0.021	0.907

Spearman’s rank correlation coefficients (r) are reported for associations between Model for End-stage Liver Disease (MELD), aspartate aminotransferase-to-platelet ratio index (APRI), Child-Pugh score (CPS), and fibrosis-4 index (FIB-4) with serum creatinine, estimated glomerular filtration rate (eGFR), and urea. Statistically significant correlations were observed only for MELD with creatinine (r = 0.333, p < 0.001) and with eGFR (r = –0.294, p = 0.001).

### Regression analyses

#### Logistic regression for KDIGO stage ≥ 3.

MELD was significantly associated with KDIGO stage ≥ 3 (B = 0.322, SE = 0.092, Wald = 12.286, p < 0.001; OR = 1.379 [95% CI: 1.152–1.651]). CPS showed a significant inverse association (B = −0.690, SE = 0.225, Wald = 9.376, p = 0.002; OR = 0.501 [95% CI: 0.322–0.780]).

No significant associations were found for APRI (p = 0.318) or FIB-4 (p = 0.364) (Table 5).

**Table 5:** Multivariable logistic regression for the prediction of severe renal dysfunction (KDIGO stage ≥ 3).

Predictor	B	SE	OR (Exp[B])	95% CI for OR	Wald χ²	p-value
**MELD**	**0.322**	0.092	**1.379**	**1.152-1.651**	12.286	**< 0.001**
**CPS**	**−0.690**	0.225	**0.501**	**0.322-0.780**	9.376	**0.002**
APRI	−0.366	0.366	0.694	0.339-1.421	0.999	0.32
FIB-4	0.121	0.133	1.129	0.869-1.466	0.825	0.36
Intercept	1.310	1.505	3.705	–	0.758	0.38

Logistic regression analysis for prediction of advanced renal dysfunction (KDIGO stage ≥ 3). Odds ratios (OR) and 95% confidence intervals (CI) are presented. MELD was positively associated with severe renal dysfunction (p < 0.001), while the Child-Pugh score (CPS) showed an inverse association (p = 0.002). APRI and FIB-4 were not significant predictors.

Abbreviations: KDIGO, Kidney Disease: Improving Global Outcomes; MELD, Model for End-stage Liver Disease; CPS, Child-Pugh score; APRI, aspartate aminotransferase-to-platelet ratio index; FIB-4, fibrosis-4 index; OR, odds ratio; CI, confidence interval; SE, standard error.

#### Linear regression for eGFR.

MELD was negatively associated with eGFR (B = −3.570, SE = 0.848, β = −0.491, t = −4.208, p < 0.001).

CPS was positively associated with eGFR (B = 5.597, SE = 2.049, β = 0.324, t = 2.732, p = 0.008).

APRI and FIB-4 were not significantly associated with eGFR (both p > 0.85) (Table 6).

**Table 6:** Multivariable linear regression predicting estimated glomerular filtration rate (eGFR) from liver function scores.

Predictor	B	SE	β (Standardized)	t	p-value	95% CI for B
**MELD**	**−3.570**	0.848	**−0.491**	−4.208	**< 0.001**	**−5.263 to −1.877**
**CPS**	**5.597**	2.049	**0.324**	2.732	**0.008**	**1.508 to 9.686**
APRI	0.319	2.100	0.075	0.152	0.880	−3.871 to 4.510
FIB-4	−0.017	1.043	−0.008	−0.017	0.987	−2.098 to 2.064
Intercept	69.982	17.613	–	3.973	< 0.001	34.836 to 105.128

Multivariable linear regression model predicting eGFR from liver function scores. Standardized β coefficients and 95% confidence intervals (CI) are presented. MELD was inversely associated with eGFR (p < 0.001), while the Child-Pugh score (CPS) showed a positive association (p = 0.008). No significant associations were observed for APRI or FIB-4.

Abbreviations: eGFR, estimated glomerular filtration rate; MELD, Model for End-stage Liver Disease; CPS, Child-Pugh score; APRI, aspartate aminotransferase-to-platelet ratio index; FIB-4, fibrosis-4 index; CI, confidence interval; SE, standard error.

### ROC analyses

**Fig 1:**
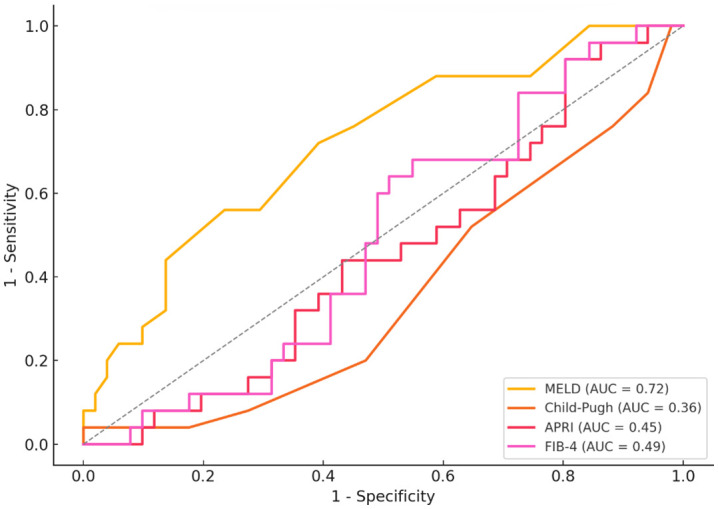
Receiver operating characteristic (ROC) curves for MELD, Child-Pugh score (CPS), APRI, and FIB-4 in predicting severe renal dysfunction (KDIGO stage ≥ 3). The figure shows ROC curves of the Model for End-stage Liver Disease (MELD; AUC = 0.72), Child-Pugh score (CPS; AUC = 0.36), aspartate aminotransferase-to-platelet ratio index (APRI; AUC = 0.45), and fibrosis-4 index (FIB-4; AUC = 0.49) for predicting severe renal dysfunction, defined as Kidney Disease: Improving Global Outcomes (KDIGO) stage ≥ 3. The diagonal dashed line represents the reference line of no discrimination.

ROC analysis showed that MELD had the highest discriminatory ability for KDIGO stage ≥ 3 (AUC = 0.718), followed by FIB-4 (AUC = 0.489), APRI (AUC = 0.453), and CPS (AUC = 0.357) (Fig 1).

The optimal MELD cutoff was 13.5 (sensitivity 56%, specificity 77%, Youden index 0.325). FIB-4 yielded a cutoff of 5.62 (sensitivity 68%, specificity 57%). APRI achieved high sensitivity (92%) but moderate specificity (80%) at a cutoff of 0.79. CPS showed the lowest performance with a Youden index of 0.040 (cutoff 13.5; sensitivity 4%, specificity 100%).

### Performance of machine learning models for CKD classification

The initial Random Forest model [28], trained with 1,000 trees, showed limited performance with an out-of-bag (OOB) error of 30%. Accuracy was 64%, and the AUC was 0.588. Specificity for KDIGO stage ≥ 3 was low (25%).

Model optimization with 50,000 trees, class weighting, and tuning of the *mtry* parameter improved performance: accuracy increased to 76%, specificity to 50%, and AUC to 0.802. Inter-rater reliability was moderate (Cohen’s kappa, κ = 0.41).

Further improvement was achieved by applying the ROSE method [25] to balance class distribution in the training set. The resulting RF model achieved 64% test accuracy, 75% specificity for KDIGO stage ≥ 3, and an AUC of 0.728.

In contrast, XGBoost models [29] trained with 500 and 5,000 trees demonstrated severe overfitting, with AUC values of 1.00 in training but only 0.490 and 0.438 in testing. Early stopping did not prevent overfitting, and specificity for KDIGO stage ≥ 3 remained low (11%), limiting their clinical utility.

**Fig 2:**
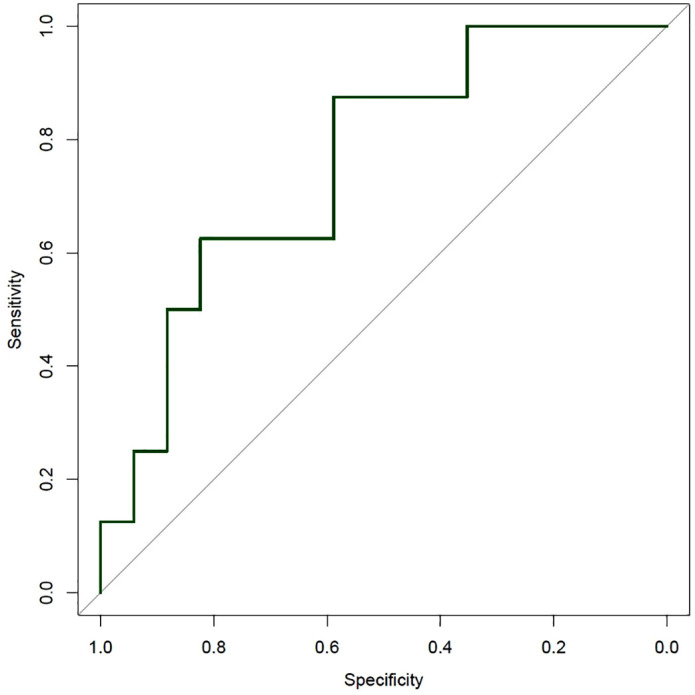
Performance of the final optimized Random Forest (RF) model for predicting severe renal dysfunction (KDIGO stage ≥ 3) in alcoholic liver cirrhosis. The ROC curve of the final RF model shows an AUC of 0.76 for predicting severe renal dysfunction, defined as KDIGO stage ≥ 3. The diagonal grey line represents the reference line of no discrimination.

The final RF model, trained with ROSE oversampling and refined feature selection, achieved 76% accuracy, 82% sensitivity for KDIGO < 3, 63% specificity for KDIGO ≥ 3, an AUC of 0.757, balanced accuracy of 72%, and moderate agreement (κ = 0.45) (Fig 2).

**Fig 3:**
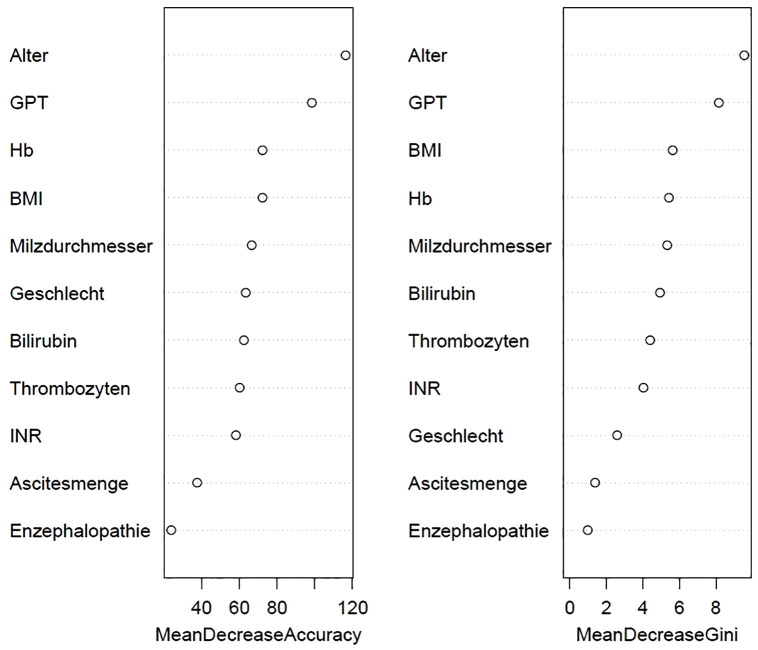
Top predictive variables for KDIGO stage ≥ 3 identified by the final Random Forest model. Variable importance plots of the final Random Forest (RF) model showing the most predictive variables for severe renal dysfunction, defined as KDIGO) stage ≥ 3. Importance is displayed as mean decrease in accuracy (left panel) and mean decrease in Gini index (right panel). Variables include age, glutamate pyruvate transaminase (GPT; alanine aminotransferase, ALT), hemoglobin (Hb), body mass index (BMI), spleen diameter, sex, bilirubin, platelet count, international normalized ratio (INR), ascites volume, and hepatic encephalopathy.

The most influential predictors in the final model were ALT, platelet count, age, INR, and BMI (Fig 3).

## Discussion

This study systematically evaluated the predictive utility of four widely used liver function scores – MELD, CPS, APRI, and FIB-4 – for identifying advanced renal dysfunction (KDIGO stage ≥ 3) in patients with alcoholic cirrhosis. In addition, supervised ML methods were applied to identify non-renal predictors that may enhance risk stratification. Our analysis yielded several novel observations: (i) an inverse association between CPS and CKD, (ii) minimal predictive value of fibrosis-based scores (APRI, FIB-4), and (iii) overfitting of XGBoost models compared with the more robust Random Forest approach. Together, these findings highlight the limitations of conventional scoring systems and the potential of ML-based models to capture broader determinants of renal dysfunction.

Among conventional scores, MELD was the strongest predictor of CKD stage ≥ 3, with significant associations in both logistic and linear regression models and an AUC of 0.718. The prevalence of CKD increased from 17% in patients with MELD ≤ 9–80% in those with MELD ≥ 20, underscoring its prognostic relevance in advanced disease. These results align with prior studies linking MELD to renal dysfunction and mortality, likely due to its inclusion of serum creatinine as a core variable [18,30–34]. However, MELD demonstrated only moderate sensitivity (56%) at the optimal cutoff, suggesting a role primarily in identifying patients who may benefit from nephrology consultation rather than in early CKD screening.

The reliance of MELD on serum creatinine introduces notable limitations, particularly in cirrhosis where sarcopenia and malnutrition can lead to falsely low creatinine and overestimated GFR [35,36]. Gender-related muscle mass differences may further affect accuracy [37,38]. Future approaches may benefit from incorporating alternative renal markers such as cystatin C or inflammatory parameters (e.g., IL-6, CRP) [39,40], and from using eGFR-based rather than creatinine-based thresholds.

Fibrosis-based scores (APRI, FIB-4) showed no significant association with renal parameters. Their low AUCs (0.453 and 0.489, respectively) and absence of meaningful correlations support the notion that CKD in cirrhosis is driven less by hepatic fibrosis and more by systemic and hemodynamic alterations [41]. Although FIB-4 has been associated with renal outcomes in metabolic or non-cirrhotic populations [19,42,43], these discrepancies likely reflect distinct pathophysiologic drivers in alcohol-associated cirrhosis, including toxic-metabolic, hemodynamic, and inflammatory factors.

The inverse association between CPS and CKD stage ≥ 3 was unexpected. CPS relies on subjective or indirect clinical variables (e.g., ascites, encephalopathy) and does not include renal markers. Patients with higher CPS may have received more intensive inpatient therapy (e.g., fluid resuscitation), potentially improving renal markers at the time of measurement. In addition, sarcopenia-related overestimation of GFR may have biased this association [35,36]. These factors illustrate the limited utility of CPS for renal risk stratification, despite its widespread use in hepatic staging [44].

ML-based models identified non-traditional predictors of renal dysfunction, including GPT, platelet count, INR, BMI, and age. The optimized Random Forest model, refined by ROSE oversampling and feature selection, achieved an AUC of 0.757 with balanced accuracy of 72%. Its performance exceeded that of conventional scores and illustrates the capacity of ML to capture multifactorial predictors of CKD. Notably, none of the top predictors were renal-specific. GPT may indicate hepatocellular injury or catabolic activity contributing to hepatorenal dysfunction [1,45], while platelet count and INR are established markers of portal hypertension and coagulopathy [46,47]. Age and BMI represent general CKD risk factors [48,49]. These findings support the integration of broader clinical markers into renal risk models for cirrhosis [6,50]. Inflammatory markers (e.g., CRP, IL-6) and renal hormones (e.g., aldosterone, renin) were not available in this retrospective dataset but may provide additional discriminatory value. Future studies should assess whether such biomarkers can further refine prediction models [39,40].

XGBoost models, despite theoretical advantages, suffered from overfitting and poor test performance (AUCs < 0.50). This highlights the importance of algorithm selection, particularly in smaller datasets, and supports the relative robustness of Random Forest in this context.

From a clinical perspective, one in three patients had CKD stage ≥ 3, emphasizing the need for systematic renal risk assessment in alcoholic cirrhosis. These patients were significantly older, had higher serum creatinine and urea levels, and more often required endoscopic therapy, consistent with prior studies linking renal dysfunction and portal hypertensive complications [6,51–53].

While our Random Forest model performed well internally, its generalizability remains to be tested. External validation in independent cohorts is essential, and transferability to other cirrhosis etiologies (e.g., viral hepatitis, NAFLD) will likely require recalibration. Importantly, the model excluded serum creatinine and GFR to avoid circularity, providing an unbiased stratification framework. Future studies should prospectively validate such models and explore integration into electronic health records or clinical decision support systems.

In summary, MELD remains the most robust conventional score for renal risk stratification in alcoholic cirrhosis. However, ML-based approaches incorporating systemic and hepatic variables offer improved diagnostic value and may enable earlier identification of at-risk individuals. Their clinical implementation will require external validation and prospective evaluation.

### Limitations

This study has several limitations. First, its retrospective single-center design may limit generalizability, particularly to non-alcoholic etiologies of cirrhosis and more diverse patient populations. Second, renal function was assessed using serum creatinine, which can underestimate impairment in patients with sarcopenia, malnutrition, or in women due to reduced muscle mass. Third, potentially relevant biomarkers – such as inflammatory markers (CRP, IL-6) and renal hormones (aldosterone, renin) – were not available in this retrospective dataset. Their absence likely restricted the scope of the models, and future prospective studies should explicitly incorporate these variables to improve predictive accuracy. Fourth, although the Random Forest model demonstrated promising internal performance, external validation in independent cohorts is required to confirm its robustness and applicability. Finally, long-term renal and survival outcomes were not assessed, which precludes prognostic interpretation beyond cross-sectional associations.

## Conclusion

Among conventional liver function scores, MELD showed the highest accuracy for identifying severe renal dysfunction (KDIGO stage ≥ 3) in patients with alcoholic cirrhosis. In contrast, CPS was inversely associated with renal dysfunction, and fibrosis-based scores (APRI, FIB-4) had no meaningful predictive value in this setting. A Random Forest–based machine learning model improved diagnostic performance by incorporating additional clinical features – such as GPT, platelet count, INR, BMI, and age – that are not represented in standard hepatic scores. These results support the notion that renal dysfunction in cirrhosis is influenced by systemic factors beyond liver disease severity.

Although the model performed well internally, external validation in independent and etiologically diverse cohorts is required before clinical application. Future studies should assess whether integrating novel biomarkers, including inflammatory markers (CRP, IL-6) and renal hormones (aldosterone, renin), can further enhance prediction and refine clinical decision-making.

## Supporting information

S1 Table Endoscopic findings and intervention requirements in the study cohort, stratified by KDIGO stage < 3 and ≥ 3. (DOCX)
