# Supplementary material for: Predicting severe renal dysfunction in alcohol-associated cirrhosis: Comparative performance of liver function scores and machine learning models
Source: PLoS One. 2025 Sep 17;20(9):e0332840. doi: 10.1371/journal.pone.0332840 (PMC12443302; doi:10.1371/journal.pone.0332840)
Supplement: S1 Table — (DOCX) [file pone.0332840.s001.docx]

**Supplementary Table S1. Endoscopic findings and intervention requirements in the study cohort, stratified by KDIGO stage < 3 and ≥ 3.**

| **Variable** | **Total (n = 131)** | **KDIGO < 3 (n = 88)** | **KDIGO ≥ 3 (n = 43)** | **p-value** | **Effect size (φ)** |
| --- | --- | --- | --- | --- | --- |
| **Presence of esophageal varices** | 125 (95.4%) | 82 (93.2%) | 43 (100%) | 0.18 | 0.15 |
| **Number of varices** |  |  |  |  |  |
| Few (1-2) | 61 (48.8%) | 35 (42.7%) | 26 (60.5%) | 0.07 | 0.16 |
| Multiple (3-4) | 64 (51.2%) | 47 (57.3%) | 17 (39.5%) | 0.05 | 0.18 |
| **Paquet variceal grading** |  |  |  |  |  |
| Grade 1 | 99 (75.6%) | 65 (73.9%) | 34 (79.1%) | 0.52 | 0.06 |
| Grade 2 | 24 (18.3%) | 17 (13.0%) | 7 (16.3%) | 0.67 | 0.04 |
| Grade 3 | 2 (1.5%) | 0 | 2 (4.7%) | **0.04** | 0.18 |
| **High-risk signs of varices** | 20 (16.0%) | 12 (14.6%) | 8 (18.6%) | 0.57 | 0.05 |
| **Prior variceal treatment** | 7 (5.3%) | 4 (4.5%) | 3 (7.0%) | 0.56 | 0.05 |
| **Need for endoscopic intervention** | 43 (32.8%) | 24 (27.3%) | 19 (44.2%) | 0.05 | 0.17 |
| **Acute variceal bleeding** | 4 (3.2%) | 4 (4.8%) | 1 (1.5%) | 0.14 | 0.13 |
| **History of prior variceal bleeding** | 13 (9.9%) | 9 (10.2%) | 4 (9.3%) | 0.87 | 0.02 |

Categorical variables are presented as counts and percentages. Effect sizes are reported as standardized mean differences (φ). Statistically significant results (p ≤ 0.05) are shown in bold.
Abbreviations: KDIGO, Kidney Disease: Improving Global Outcomes.
